# Supplementary material for: Identification of Bioactive Phytochemicals from Six Plants: Mechanistic Insights into the Inhibition of Rumen Protozoa, Ammoniagenesis, and α-Glucosidase
Source: Biology (Basel). 2021 Oct 18;10(10):1055. doi: 10.3390/biology10101055 (PMC8533169; doi:10.3390/biology10101055)
Supplement: Supplementary file 1 [file biology-10-01055-s001.zip › biology-1362845-supplementary.pdf]

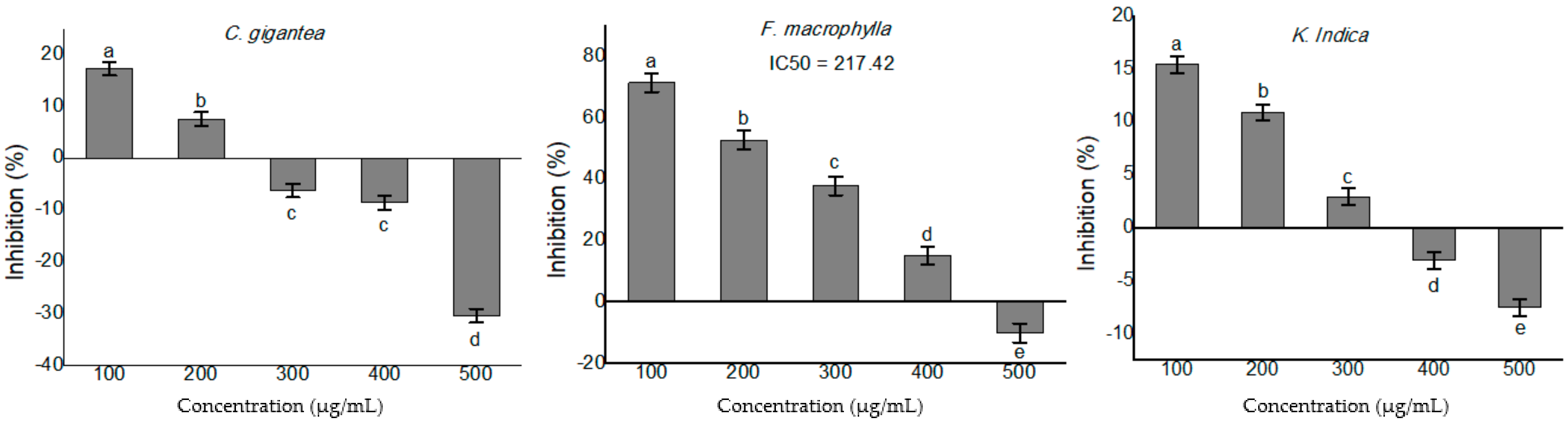

Figure. S1: Inhibition of α-glucosidase activities by the extract of *C. gigantea, F. macrophylla,* and *K. indica*. The extracts of these plants produced unexpected results with the inhibition diminishing, even α-glucosidase activity increasing, when the extract dose increased. Only the extract of *F macrophylla* (leaves) had calculated IC_{50} value. The extracts of *Portulaca Oleracea* and *Flemingia macrophylla* (roots) samples were not tested as they were lost. Means with different lower-case letters on the same graph differ ($P < 0.05$).
